# Analytical Modeling and Validation of a Preloaded Piezoceramic Current Output

**DOI:** 10.3390/mi12040353

**Published:** 2021-03-25

**Authors:** Bin Zhang, Hongsheng Liu, Dezhi Li, Jinhui Liang, Jun Gao

**Affiliations:** 1 School of Mechanical, Electrical and Information Engineering, Shandong University, Weihai 204209, China; liuhongsheng96@mail.sdu.edu.cn (H.L.); liangjinhui@mail.sdu.edu.cn (J.L.); shdgj@sdu.edu.cn (J.G.); 2 Department of Engineering Mechanics, Zhejiang University, Hangzhou 310027, China; dezhi.li@zju.edu.cn

**Keywords:** current output, multivariate model, piezoelectric coefficient, stimulation parameter consideration

## Abstract

Energy harvesting using piezoceramic has drawn a lot of attention in recent years. Its potential usage in microelectromechanical systems is starting to become a reality thanks to the development of an integrated circuit. An accurate equivalent circuit of piezoceramic is important in energy harvesting and the sensing system. A piezoceramic is always considered to be a current source according to empirical testing, instead of the derivation from its piezoelectric characteristics, which lacks accuracy under complicated mechanical excitation situations. In this study, a new current output model is developed to accurately estimate its value under various kinds of stimulation. Considering the frequency, amplitude and preload variation imposed on a piezoceramic, the multivariate model parameters are obtained in relation to piezo coefficients. Using this model, the current output could be easily calculated without experimental testing in order to quickly estimate the output power in energy harvesting whatever its geometric shape and the various excitations.

## 1. Introduction

Wireless sensor networks (WSNs) are widely used in the industry, for military applications and smart homes, to monitor the operating conditions or environmental status. Though the data can be transmitted wirelessly, the batteries need to be recharged or replaced periodically. The strong demand for self-powered wireless sensor network nodes encourages researchers to study different energy harvesting mechanisms: solar, piezoelectricity, thermoelectricity, electromagnetism and so on. A piezoceramic can be used to transfer the ubiquitous vibration into electricity so as to prolong the battery lifespan or even provide life-long power for sensor nodes.

By designing a self-powered electronic gadget, lots of factors, such as the environmental parameters (energy type, intensity, characteristics, etc.), energy consumption, energy conversion mechanism, transfer efficiency, etc., should be carefully studied or coupled. Solar energy is abundant and of high efficiency, especially in an outdoor situation, but it is susceptible to the weather. Otherwise, vibration is a promising energy source for embedded self-powered devices [1]. The wireless sensor nodes’ power consumption varies from nanowatts to watts, since energy dissipation is a key issue for all kinds of WSNs. Considering the available environmental vibration energy density and achievable energy conversion efficiency are attained, a piezoelectric energy harvesting system can be used in cases lower than milliwatt or microwatt depending on its periodic or event-responded monitoring in an optimized WSN. Kim et al. [2] have reviewed the WSN energy consumption balancing (ECB) and categorized the powered consumption influencing factor including the node distribution, base station option and application type. Improvement measures can be introduced into the WSNs to maximize the use of energy to prolong node lifespan [2,3].

As a promising mechanism for self-powered sensor nodes, piezoelectric energy harvesting (PEH) has been studied by a number of researchers for the last two decades. Umeda [4], Shu [5], Yang [6] and others have studied the efficiency of mechanical energy transformation to electrical energy, which is an important indicator to optimize the coupling factors and PEH structure design. The lumped parameter model along with the Euler–Bernoulli beam theory is always used to model the PEH system, including the one degree of freedom (1DOF) [7] or 2DOF [8] vibration system. As the piezoceramic is considered a current source, the electric displacement is simplified to a linear relationship with external stress. Under an actual scene, the piezoceramic output changes along with the preload, frequency, etc. The output power is limited by the high impedance in a piezoceramic. A high-precision oscillation circuit can be used to enhance the output performance of the piezoceramic. Lots of efforts have been put on the interface circuit, which is a most important technique and can significantly improve the electricity energy extracted from the piezoceramic when accurately coupled. Lallart et al. [9,10,11,12,13,14] have studied the nonlinear interface circuit, as well as low-voltage, energy storage, wide bandwidth and self-powered situations.

Note that a piezoceramic is usually considered to be a current source paralleled with a capacitor, and its current output is essential to design a piezoelectric energy source when a micro-electro-mechanical systems (MEMS) device is fabricated in small size. The optimal design of the piezo source is fundamental in the self-powered WSN node, for example, the selection of piezoceramic type, geometric size, stimulation stress and so on. In different piezoelectric energy harvester structures, such as a bistable generator [15], tri-stable energy harvester [16], spring pendulum oscillator [17], clamped piezo-stack [18], etc., and a horizontal cantilever beam with the consideration of gravity, the piezoceramic is in a preloaded status which will affect its current output. It is necessary to establish a model that includes the common variables during the deployment of piezoceramic devices.

Liao [19] developed a reduced model to predict and optimize a cantilever beam energy harvesting system and it is efficiency to get an optimal resistance in beam-like structures. The current output, accompanied by its geometric size, stimulation characteristic and piezo coefficients is studied in this paper to further simplify the rapid prototyping energy source design. Regardless of whether the base structure is a cantilever beam, cymbal type or others, this model, derived from the stress distribution in a piezoceramic, gives an accurate estimation of the current output. Since an efficient energy harvesting system is the optimum design of different components, the selection of interface circuit elements is particularly important. Morel et al. studied the influence of the resistive, capacitive and inductive behavior in a piezoelectric energy harvesting system and derived a simplified model [20], and they also manufactured an extra low energy consumption integrated interface to achieve >91% efficiency of energy harvesting under shocks and 94% under periodic excitations [21]. Their studies have the potential to improve the interface-circuit parameter coupling for the current output model. The summary from Brenes et al. [22] facilitates the interface circuit implementation and helps to achieve the maximum power output in the piezoelectric energy harvesting. In the former studies, Ducharne and other researchers [23,24,25,26] have studied the nonlinearity in piezoceramic and its application in energy harvesting. The proposed model will include the nonlinearities as well.

In the next section, the current output model is established. Section 3 is the experimental validation of the model under different preloads, frequencies and excitation amplitudes. Conclusions will be drawn in the final section.

## 2. Model and Theory

### 2.1. Electromechanical Coupling Model

**Figure 1:**
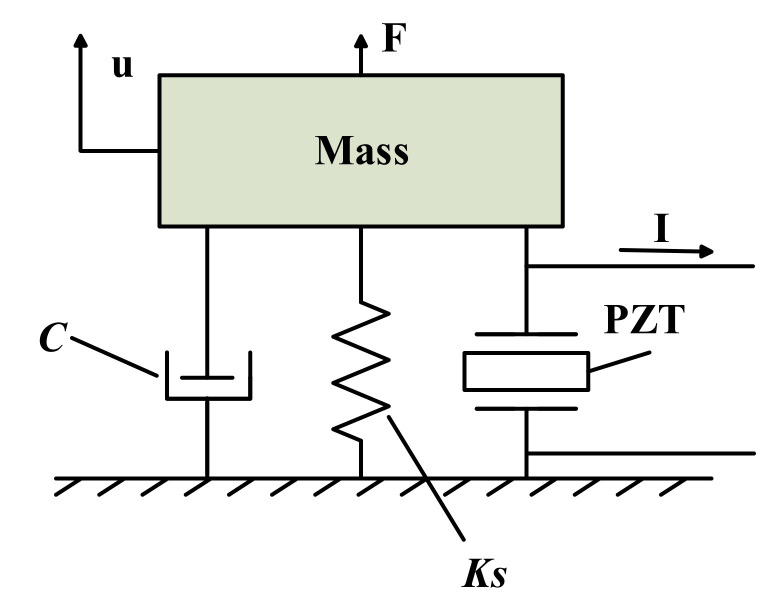
Electromechanical coupling model.

The electromechanical coupling model is established to combine electrical quantity with mechanical quantity [27]. In order to facilitate the analysis, this paper uses a single-degree-of-freedom case for the mathematical model [28]. The electromechanical coupling model of the vibration energy harvesting system can be represented by the “mass + damping + spring”, as shown in Figure 1.

The mechanical loss is equivalent to the viscous damper *C*, while *M* is the equivalent mass of the block, *K*_S_ is the equivalent stiffness of the structure, *F* is the external excitation and *u* is the displacement of the structure. The governing equations of motion (1) can be obtained from Newton’s law [20,22,29].

(1) $$\left\{\begin{array}{l} M\ddot{u}+C\dot{u}+K_{S}u=\sum F_{i}\\ \sum F_{i}=F+F_{P} \end{array}\right.$$

Among them, Σ*F*_i_ is the external force acting on the structure, including the external excitation *F* and the reaction force *F*_P_ of piezoelectric plates.

For the piezoelectric system in this paper, the boundary conditions are short circuit and mechanically free. The piezoelectric equations are shown in Equation (2) [30], where *T* is the stress of the piezoelectric plate, *S* is the strain of the piezoelectric plate, *E*_3_ is the electric field strength, *D* is the electric displacement, *s*^E^_33_ is the short-circuit elastic compliance, *d*_33_ is the piezoelectric constant and *ε*^T^_33_ is the constant stress dielectric permittivity.

(2) $$\left\{\begin{array}{c} S_{3}=s_{33}^{E}T_{3}-d_{33}E_{3}\\ D_{3}=d_{33}T_{3}+\varepsilon_{33}^{T}E_{3} \end{array}\right.$$

Because [31,32]

(3) $$E_{3}=-\frac{V}{L},S=\frac{u}{L},I=A\frac{dD}{dt},F_{P}=AT$$

*V* and *I* represent the voltage of the piezoelectric patch and the current flow, respectively. *L* and *A* represent the thickness and surface area of the piezoelectric patch, respectively. *F*_P_ is the reaction force of the piezoelectric patch to the base structure. Equation (3), combined with Equation (2), can derive Equation (4) [20,22,30,31,32].

(4) $$\left\{\begin{array}{l} F_{P}=K_{\mathrm{PE}}u+\alpha V\\ I=\alpha \dot{u}-C_{P}\dot{V} \end{array}\right.$$

*K*_PE_ is equivalent to the short-circuit stiffness of the piezoelectric block, *C*_P_ is the equivalent capacitance of the piezoelectric block and *α* is the stress factor [31,32].

(5) $$K_{\mathrm{PE}}=\frac{c_{33}^{E}A}{L},C_{P}=\frac{\varepsilon_{33}^{S}A}{L},\alpha =\frac{e_{33}A}{L},\;K_{\mathrm{PD}}=\frac{s_{33}^{D}A}{L}=\frac{K_{\mathrm{PE}}}{1-k_{t}^{2}}$$

(6) $$k_{t}^{2}=\frac{e_{33}^{2}}{\varepsilon_{33}^{E}c_{33}^{E}}=\frac{e_{33}^{2}}{\varepsilon_{33}^{S}c_{33}^{E}+e_{33}^{2}}$$

A global stiffness for both the mechanical structure and the piezoelectric disk can be defined as (5) when a piezoceramic is on short circuit (*K*_PE_) and on an open circuit (*K*_PD_). The global electromechanical coupling factor *k*_t_ is then expressed in Equation (6). As the solution of this study is to estimate the short-circuit current, *K*_PE_ is taken into account here.

According to the law of conservation of energy, the input energy of the whole system is assumed as *E*. In this system, the input energy is transformed into kinetic energy, elastic potential energy, loss and piezoelectric energy, respectively. Its expression is shown in Equation (7):

(7) $$E=E_{P}+E_{K}+E_{D}+E_{E}$$

*E*_D_ is mainly composed of heat loss *Q*_C_ and dielectric loss *Q*_D_. As the vibration generates small polarization, and the loss angle tan*δ* is relatively small at low voltage, *Q*_D_ can be neglected. Its *Q*_C_ is mainly caused by ESR (Equivalent series resistance). The expression is as follows:

(8) $$\begin{array}{l} Q_{C}=\int I_{\mathrm{RMS}}^{2}\times ESRdt\\ ESR=\frac{X_{C}}{Q}=\frac{1}{2\pi fC_{P}Q} \end{array}$$

*I*_RMS_ is the effective value of current output, *X*_C_ is capacitive reactance, and *Q* is the quality factor of capacitance.

Multiply *u* on both sides of the Equation (1) and integrate *t*. From Hamilton’s Principle, the Equation (9) can be obtained [21].

(9) $$\int F\dot{u}dt=\frac{1}{2}M{\dot{u}}^{2}+\frac{1}{2}K_{\mathrm{PE}}u^{2}+\int C{\dot{u}}^{2}dt+\int \alpha V\dot{u}dt$$

Equation (9) can represent the existence of energy in the whole system, and the physical meanings represented by each of them are shown in Table 1 [21].

As can be seen from the table, $\int \alpha V\dot{u}dt$ is the energy we need. By multiplying the second sub-formula of Equation (4) by *V* and integrating *t*, the Equation (10) can be obtained.

(10) $$\int VIdt=\int \alpha V\dot{u}dt-\frac{1}{2}C_{P}V^{2}$$

$\frac{1}{2}C_{p}V^{2}$ is the energy stored in the equivalent capacitance. ∫VIdt is the real energy flowing into the subsequent acquisition circuit and used for the load.

### 2.2. Current Output Model

Considering the dynamic characteristics of the piezoelectric ceramic and the external excitation force, Equation (1) can be expressed as follows:

(11) $$M\ddot{u}+C\dot{u}+K_{S}u=F_{0}\sin\omega t$$

What mainly matters in forced vibration is the steady-state vibration, whose solution is:

(12) $$\left\{\begin{array}{l} u(t)=\frac{F_{0}}{M\;\sqrt{{\left(\omega_{n}^{2}-\omega^{2}\right)}^{2}+{\left(\frac{C\omega }{M}\right)}^{2}}}\sin(\omega t+\theta )\\ \theta =\arg\tan\frac{C\omega }{M(\omega_{n}^{2}-\omega^{2})} \end{array}\right.$$

The natural frequency of cylindrical piezoceramic (PZT5, Φ6 × 5 mm) is extremely high compared with the exciting frequency, and the capacitor of the piezoceramic is 0.25 nF. The steady state solution can be simplified as follows:

(13) $$\left\{\begin{array}{l} u(t)=\frac{F_{0}\sin\omega t}{M(\omega_{n}^{2}-\omega^{2})}\\ \omega_{n}=\sqrt{\frac{K_{S}}{M}} \end{array}\right.$$

Therefore, the voltage at both ends of the PZT *V*(*t*) is:

(14) $$\left\{\begin{array}{l} V(t)=\frac{d_{33}LT_{3}(t)}{\varepsilon_{r}\varepsilon_{0}(1-\frac{\omega^{2}}{\omega_{n}^{2}})}\\ T_{3}=T_{0}\sin\omega t\\ T_{0}=\frac{F_{0}}{A} \end{array}\right.$$

Its current output *I*(*t*) can be obtained as follows:

(15) $$I(t)=C_{P}\frac{dV}{dt}=C_{P}\frac{d_{33}\omega L}{\varepsilon_{r}\varepsilon_{0}(1-\frac{\omega^{2}}{\omega_{n}^{2}})}T_{0}\cos\omega t$$

**Figure 2:**
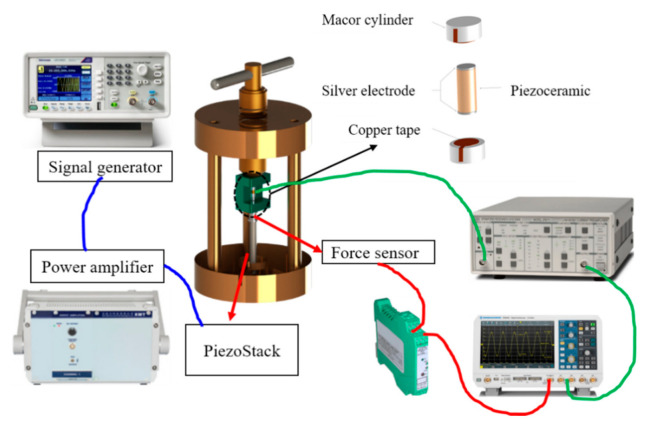
Configuration of the experimental platform.

In order to consider the effect of preloading *P*_pre_ on the current output, the bias stress parameter *β* is introduced considering the mechanical conduct and dynamic factor, mainly affected by the contact type. As shown in Figure 2, the piezoceramic is compression-fixed block by block, in different material. *P*_Pre_ is necessary to keep these blocks together. The excitation amplitude is required to be smaller than the magnitude of *P*_Pre_ to assure the piezoceramic is in good mechanical contact. The equivalent stress applied to the piezoceramic should consider both the variation and the preload. Here, in this case, the *β* should always be positive. However, in a cantilever-beam-like structure, it can be either positive or negative, as a piezoceramic can be compressed or stretched. The stress *T* is expressed as *T’*:

(16) $$T^{\prime }=T_{0}+\beta T_{0}$$

In this experiment setup, the *β* value is expressed as:

(17) $$\beta =0.1687\times P_{\mathrm{pre}}+0.0122$$

Therefore, the final short circuit current of the system is:

(18) $$\begin{array}{ll} I(t) & =C_{P}\frac{dV}{dt}=C_{P}\frac{d_{33}\omega L}{\varepsilon_{r}\varepsilon_{0}(1-\frac{\omega^{2}}{\omega_{n}^{2}})}T_{0}(1+\beta )\cos\omega t\\ & =\frac{2\pi fd_{33}\varepsilon_{33}^{S}A}{\varepsilon_{r}\varepsilon_{0}(1-\frac{4\pi^{2}f^{2}LM}{c_{33}^{E}A})}T_{0}(1+\beta )\cos2\pi ft \end{array}$$

Since the stress distribution is easily analyzed by the finite element method, a piezoceramic dedicated to be used in an energy harvesting system is designed accordingly. By the use of Equation (18), one can easily calculate the current output of the piezoceramic so as to select an appropriate energy extraction circuit and evaluate the satisfaction of energy supply [19,20].

## 3. Experimental Verification of Current Output Model

**Figure 3:**
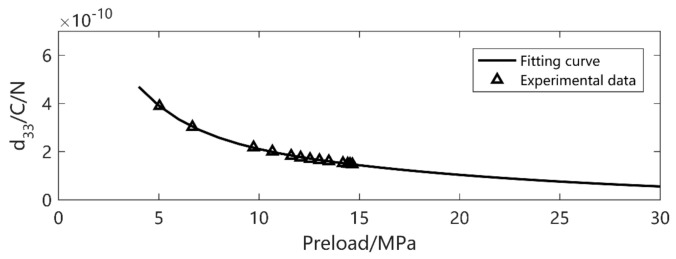
*d*_33_ value versus preload.

In order to verify the accuracy of the current output model, a preload test platform is built to measure the experimental data. Figure 2 shows the schematic diagram of the experimental connections. The platform consists of a signal generator (TEKTRONIX, AFG1062) which controls the power amplifier (COREMORROW), the oscilloscope (ROHDE&SCHWARZ, RTB2004), the force sensor and the current amplifier, connected as follows:

Then, the piezoceramic is excited under different amplitudes (5–70 MPa), preload pressure (5–45 MPa) and frequencies (5–70 Hz). Though the piezoelectric constitutive equations are always correct in any case, the piezo coefficients vary according to the external excitation. Under small excitation, *d*_33_ variation is mainly due to the preload. With the increase of preload, *d*_33_ decreases sharply at the beginning and then trends to be steady. Under the 5 MPa excitation, the preload varies. The experimental data and fitting figure are shown in Figure 3.

**Figure 4:**
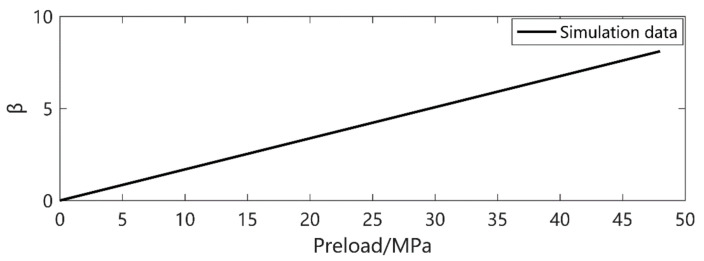
The adjustment parameter *β* value.

The curve of the adjustment parameter *β* is shown in Figure 4.

**Figure 5:**
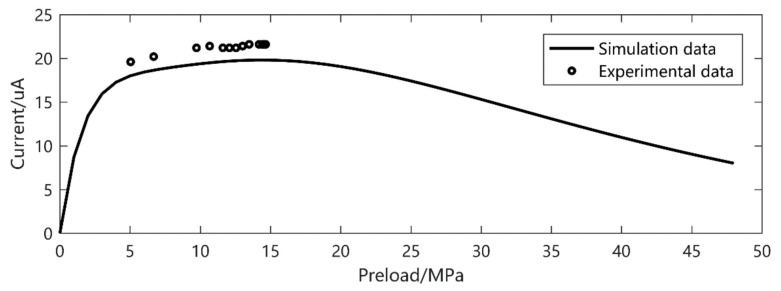
Frequency 30 Hz, excitation 11.76 MPa current output.

**Figure 6:**
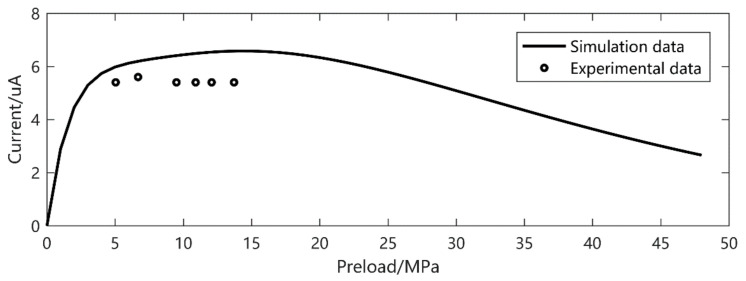
Frequency 20 Hz, excitation 5.86 MPa current output.

**Figure 7:**
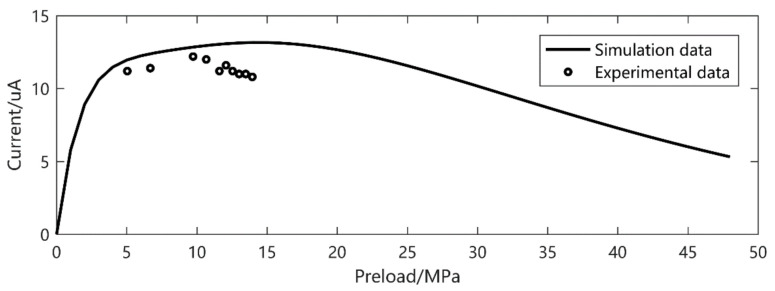
Frequency 40 Hz, excitation 5.86 MPa current output.

To verify the accuracy of the model under different excitation levels, the current output results under different frequencies, preload and excitation amplitudes were experimentally verified. When the excitation frequency is 30 Hz and the excitation amplitude is 11.76 MPa, the current output is shown in Figure 5. In another series of experiments, when the excitation amplitude is 5.86 MPa and the frequency is 20 Hz and 40 Hz, the current output is shown in Figure 6 and Figure 7, respectively.

**Figure 8:**
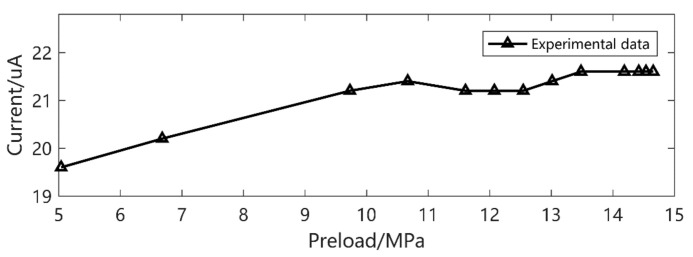
Frequency 30 Hz, excitation 11.72 MPa current output.

It is easy to see that, despite the *d*_33_ and *β* derived from the low preload and excitation, the current output gives a precise prediction, especially at higher amplitude. The prediction error is about 20% in small output magnitude. We can also see that there is a double peak phenomenon according to Figure 8 at 30 Hz, 5–15 MPa preload and 11.72 MPa excitation. This gives a strong verification of what we have found in [26].

## 4. Conclusions and Discussion

In this study, a current output model of piezoceramic is proposed by the use of piezoelectric parameters considering the electromechanical coupling. With this model, one can easily estimate the short circuit current under certain variations of mechanical excitation. By the integration of time, the electric charge can be obtained, and then the total power can be calculated. Without lots of measurements and testing, the geometric values of a piezo source would be confined, when the environmental vibration is given or analyzed by FEM. Moreover, the *d*_33_ value variation tendency is obtained on the basis of experimental data. This provides a guidance for considering the nonlinearity of piezo coefficients. Subsequently, piezoelectric ceramics can be equivalent to the electrical form of parallel connection between the current source and capacitor, which provides important support in the field of vibration energy harvesting.

Now, one can predict the total power generated by a piezoceramic under specified circumstances. Due to the experiment setup limitation, a high frequency and amplitude cannot be achieved. The analytical model gives a prediction of the piezoceramic behavior. Future studies will be concentrated on the coupling between the interface circuits to enhance energy extraction from the piezoceramic.

## Figures and Tables

**Table 1:** Energetic terms definitions.

Energy Symbol	Expression	Definition
*E*	$\int F\dot{u}dt$	System input energy
*E*_P_	$\frac{1}{2}K_{\mathrm{PE}}u^{2}$	Elastic potential energy
*E*_K_	$\frac{1}{2}M{\dot{u}}^{2}$	System kinetic energy
*E*_D_	$\int C{\dot{u}}^{2}dt$	Electrical loss
*E*_E_	$\int \alpha V\dot{u}dt$	Converted energy
